# Dermal Delivery of Diclofenac Sodium—In Vitro and In Vivo Studies

**DOI:** 10.3390/pharmaceutics14102106

**Published:** 2022-10-01

**Authors:** Fotis Iliopoulos, Choon Fu Goh, Tasnuva Haque, Annisa Rahma, Majella E. Lane

**Affiliations:** 1 Department of Pharmaceutics, UCL School of Pharmacy, 29–39 Brunswick Square, London WC1N 1AX, UK; 2 Discipline of Pharmaceutical Technology, School of Pharmaceutical Sciences, Universiti Sains Malaysia, Minden 11800, Penang, Malaysia; 3 Pharmaceutics Department, School of Pharmacy, Institut Teknologi Bandung, Bandung 40132, Indonesia

**Keywords:** confocal Raman spectroscopy, diclofenac, in vitro–in vivo correlation, skin delivery, tape stripping, topical formulations

## Abstract

Previously, we reported the use of confocal Raman spectroscopy (CRS) as a novel non-invasive approach to determine drug disposition in the skin in vivo. Results obtained by CRS were found to correlate with data from the well-established in vitro permeation test (IVPT) model using human epidermis. However, these studies used simple vehicles comprising single solvents and binary or ternary solvent mixtures; to date, the utility of CRS for monitoring dermal absorption following application of complex marketed formulations has not been examined. In the present work, skin delivery of diclofenac sodium (DFNa) from two topical dermatological drug products, namely Diclac^®^ Lipogel 10 mg/g and Primofenac^®^ Emulsion gel 1%, was determined by IVPT and in vivo by both CRS and tape stripping (TS) methodologies under similar experimental conditions. The in vivo data were evaluated against the in vitro findings, and a direct comparison between CRS and TS was performed. Results from all methodologies showed that Diclac promoted significantly greater DFNa delivery to the skin (*p* < 0.05). The cumulative amounts of DFNa which permeated at 24 h in vitro for Diclac (86.5 ± 9.4 µg/cm^2^) were 3.6-fold greater than the corresponding amounts found for Primofenac (24.4 ± 2.7 µg/cm^2^). Additionally, total skin uptake of DFNa in vivo, estimated by the area under the depth profiles curves (AUC), or the signal intensity of the drug detected in the upper stratum corneum (SC) (4 µm) ranged from 3.5 to 3.6-fold greater for Diclac than for Primofenac. The shape of the distribution profiles and the depth of DFNa penetration to the SC estimated by CRS and TS were similar for the two methods. However, TS data indicated a 4.7-fold greater efficacy of Diclac relative to Primofenac, with corresponding total amounts of drug penetrated, 94.1 ± 22.6 µg and 20.2 ± 7.0 µg. The findings demonstrate that CRS is a methodology that is capable of distinguishing skin delivery of DFNa from different formulations. The results support the use of this approach for non-invasive evaluation of topical products in vivo. Future studies will examine additional formulations with more complex compositions and will use a wider range of drugs with different physicochemical properties. The non-invasive nature of CRS coupled with the ability to monitor drug permeation in real time offer significant advantages for testing and development of topical dermatological products.

## 1. Introduction

The therapeutic efficacy of topical dermatological drug products is characterized by the rate and the extent that the active component reaches the site of action inside the skin, i.e., the stratum corneum (SC), the viable epidermis or the deeper cutaneous tissues. However, probing localized drug disposition in vivo has been historically challenging, and to date, only limited methods are available for such determinations [1,2]. Generally, in vivo dermal absorption studies in human subjects are considered to be the gold-standard experimental model, and data from such studies are normally used for the evaluation of topical and transdermal delivery systems [3,4].

Tape-stripping (TS) has been extensively explored as a technique to estimate in vivo dermatopharmacokinetics (DPK) for many years [5,6]. TS uses adhesive tapes to collect successive layers of the SC following topical application of formulations and subsequently quantify the drug content in each layer of stripped skin. TS has been combined with analytical techniques to measure the penetration of chemicals to the SC over time and evaluate the efficacy of topical dermatological dosage forms [2,7]. This method can provide useful information about the distribution of topically applied substances in the skin; however, TS has been shown to be heavily influenced by a number of experimental variables, including the type of tape used, the pressure applied by the operator, the duration of the pressure, the contact time, and the velocity of tape removal, as well as the formulation components [8,9,10,11]. This reported variability of TS has resulted in contradictory results from different laboratories and has consequently led the United States Food and Drug Administration (FDA) to withdraw a draft guidance that had previously described TS as a methodology for topical bioequivalence/bioavailability determinations. The guidance withdrawal was based on substantial doubt by the agency regarding the reproducibility of the method, and also on the fact that TS could only sample part of the SC and not deeper skin layers [12]. An additional limitation of the TS approach is that it cannot discriminate between a drug that is in solution, therefore therapeutically available, and a drug that may have crystallized out of the vehicle and be deposited on the skin and/or in the lipid inter-cellular domains inside the SC [13]. Drug crystallization is known to have significant implications for topical and transdermal delivery; therefore TS results may overestimate the formulation performance [14]. Although extensive efforts have been made to improve the TS study design toward a standardized protocol, the development of novel reproducible and reliable methods to monitor cutaneous transport of active agents has been a major research focus in recent years [2].

Confocal Raman spectroscopy (CRS) is an optical method that combines spontaneous Raman scattering emission with a confocal signal collection scheme to enable data acquisition with high spatial resolution. CRS is non-invasive and has been used in skin research for several applications, e.g., to detect and profile endogenous ingredients, to estimate SC thickness, and to determine skin hydration levels [15,16,17,18,19,20]. Additionally, CRS has been used to monitor the cutaneous transport of topically applied compounds in vivo in real time [21,22]. In 2013, Mateus and co-workers introduced CRS as a novel and non-invasive DPK methodology for the evaluation of topical pharmaceutical formulations [23]. In this study, the skin disposition of ibuprofen was determined in vivo by CRS following infinite dose application (95 µL/cm^2^) of saturated solutions of the drug in propylene glycol (PG), PG:water (50:50, *v*/*v*), and PG:water (75:25, *v*/*v*) mixtures. CRS results were compared with data that had been previously obtained by a different laboratory using TS under similar experimental conditions [24]. The comparison of the two methods was based on the diffusion parameter values for the formulations tested, estimated by fitting the depth profiles to a solution of Fick’s second law of diffusion [25], as well as on the shape of the distribution profiles across the SC. Overall, the CRS results were in accordance with TS data, suggesting that CRS was a valid technique to monitor the transport of drugs across the SC. Since then, the potential of CRS for measuring skin disposition has been further investigated in a number of studies that compared CRS results against data obtained from the well-established in vitro permeation test (IVPT) model [26,27,28]. IVPT has been shown to correlate well with in vivo results for many active agents, and the reliability of this model in assessing topical and transdermal delivery has been reported in numerous studies over the years [4,29,30,31,32].

Despite the reported potential of CRS for DPK determinations, most studies so far have used simple vehicles comprising single solvents, binary or ternary solvent mixtures, and the utility of CRS for monitoring dermal absorption following applications of complex formulations is yet to be established. The presence of additional components in commercially available products may result in spectral overlap in the acquired Raman signal, thereby increasing the complexity of signal processing for identifying the compound of interest. Additionally, to date, only a limited number of drugs has been examined by CRS in vivo, e.g., ibuprofen, salicylic acid, and flufenamic acid [23,26,33]. The aims of the present proof-of-concept study were therefore to: (i) determine the human skin disposition of diclofenac sodium (DFNa) from two commercially available gel formulations in vivo, using both CRS and TS; (ii) investigate the human skin permeation of DFNa from these formulations by the IVPT method under the same experimental conditions; and (iii) explore possible correlations between the various methodologies. DFNa was selected as a model drug for this study because it has been used in a wide range of branded and generic dermal formulations for many years. DFNa is a non-steroidal anti-inflammatory drug (NSAID), and it is typically used as an analgesic in the treatment of painful inflammatory musculoskeletal conditions and osteoarthritis. Topical DFNa products are additionally used for the management of actinic keratosis, a premalignant skin condition that is characterized by intraepidermal proliferation of dysplastic keratinocytes [34,35].

## 2. Materials and Methods

### 2.1. Materials

Diclofenac sodium (DFNa) was purchased from AK Scientific Inc. (Union City, CA, USA). Acetonitrile, trifluoroacetic acid (TFA), and High-Performance Liquid Chromatography (HPLC) grade water were purchased from Fischer Scientific (Loughborough, UK). Standard D-Squame^®^ tapes (2.2 cm in diameter, area 3.8 cm^2^) were obtained from CuDerm Corporation (Dallas, TX, USA). Phosphate-buffered saline (PBS) tablets were purchased from Oxoid Limited (Cheshire, UK). The DFNa-containing formulations Diclac^®^ Lipogel 10 mg/g (Sandoz Pharmaceuticals, Basel, Switzerland) and Primofenac^®^ Emulsion gel 10 mg/g (Streuli Pharma) were purchased from local pharmacies in Spain.

### 2.2. Methods

#### 2.2.1. HPLC Analysis

The amount of DFNa in all samples was determined using the previously reported HPLC method [36,37]. This method was validated according to the ICH guideline Q2(R1) [37,38]. The mobile phase consisted of 70% acetonitrile, 30% water, and 0.1% TFA. The lower limits of detection (LOD) and quantification (LOQ) for DFNa were 0.1 and 0.5 µg/mL, respectively.

#### 2.2.2. In Vitro Permeation Test (IVPT) Studies

IVPT studies were conducted using vertical glass Franz-diffusion cells following the OECD guidelines [39,40]. The membrane used was human epidermis prepared by heat separation according to procedures described previously [28,41]. The diffusion area of the donor chamber was ~1 cm^2^, accurately measured for each cell individually using an electronic digital micrometer (Fisher Scientific, Leicestershire, UK). The experiments were conducted in a temperature-controlled water bath (Grant Instruments, Cambridge, UK) equipped with a submersible magnetic stir plate (Variomag^®^ Telesystem, Thermo Fisher Scientific, Waltham, MA, USA). Freshly prepared PBS, pH = 7.3 ± 0.2, was used as the receptor solution. A Teflon^®^-coated magnetic stir bar in the receptor compartment ensured uniform mixing of all components in the solution throughout the experiment. The cells were placed in a water bath for approximately 30 min, and once the skin temperature had equilibrated to 32 ± 1 °C, a dose of 95 µL of the formulations was applied to the skin surface. Prior to the permeation experiments, the integrity of human skin in each Franz cell was examined by measuring the impedance of the skin according to procedures reported previously [42]. The formulations tested were Diclac^®^ Lipogel 10 mg/g (Diclac) and Primofenac^®^ Emulsion gel 10 mg/g (Primofenac). The qualitative composition of the formulations is shown in Table 1 [43]. The number of replicate experiments was *n* = 5.

#### 2.2.3. Confocal Raman Spectroscopy

Raman measurements were obtained with a Model 3510 SCA Skin Analyser Raman spectrometer (RiverD International B.V., Rotterdam, The Netherlands). This system comprises two fibre-coupled diode pumped lasers of two different wavelengths: 690 and 785 nm. These wavelengths were used to record spectra in the high wavenumber (HWN) (2500–4000 cm^−1^) and the fingerprint (FP) (400–1800 cm^−1^) region, respectively. The instrument was calibrated on the day of the experiment, as described elsewhere [28,44]. The Raman spectrum of the active agent was acquired according to procedures reported previously [26,44]. Briefly, a solution of 200 mg/mL DFNa in propylene glycol (PG) was prepared, and the spectrum of the sample was calculated as the average of 10 frames taken sequentially, with a 10 s exposure time per frame. The Raman spectrum for the neat PG solvent was additionally measured under the same conditions. The reference drug spectrum was subsequently acquired by subtracting the solvent spectrum from the spectrum of the DFNa-containing PG solution.

For the measurements, an application site measuring 4 × 4 cm^2^ was delineated on the volar forearm of two Asian volunteers (1 male, 1 female, age range: 28–31 years old). Prior to application of formulations, control measurements in both FP and HWN regions were carried out. The FP readings served as the baseline, and the HWN measurements were used for the calculation of SC thickness [16]. Infinite doses, 95 µL/cm^2^, of DFNa formulations were applied to the marked areas, and the skin was subsequently occluded with Parafilm^®^ and Tegaderm^®^ film. The formulations tested were Primofenac and Diclac, as for the IVPT studies. After 30 min, any excess of formulation was removed from the skin surface using Kimberly Clark^®^ tissue paper, and scans in the FP region were carried out with a 10 s exposure time and 4 µm steps to a final depth of 28 µm. For the HWN measurements, a 0.5 s exposure time and 2 µm steps were used to a final depth of 34 µm.

#### 2.2.4. Tape Stripping

TS was performed according to procedures previously described in the literature [45,46]. Briefly, a control area was marked on the volar forearm, very close to the application area. Each application site measured 4 × 4 cm^2^, and infinite doses (95 µL/cm^2^) of the formulations, Diclac and Primofenac, were applied, as for the CRS studies. The application sites were subsequently occluded with Parafilm^®^ and Tegaderm^®^ film. Thirty minutes after application, any excess formulation was removed with Kimberly Clark^®^ tissue paper, and a D-Squame^®^ tape (CuDerm Corporation, Dallas, TX, USA) was applied to the investigated site with a standardised D-Squame^®^ pressure instrument of 225 g/cm^2^ for 5 s. The tape was then removed and placed in a D-Squame^®^ Disc Carrier (CuDerm Corporation, Dallas, TX, USA). This procedure was repeated on the same test site to collect 20 consecutive tapes. The tapes were subsequently placed into Eppendorf^®^ tubes with 1 mL of methanol, and tubes were left for 16 h in an orbital shaker at 32 °C for drug extraction. The tubes were centrifuged at 32 °C at 12,000 rpm for 15 min in an Eppendorf 5415R centrifuge (Eppendorf, Hamburg, Germany), and the amounts of DFNa were subsequently determined by HPLC. The DFNa extraction method was validated in previous work by spiking tape-stripped samples of untreated SC with known amounts of DFNa in various vehicles, and total drug recovery was found to exceed 95% [37].

#### 2.2.5. Data Analysis

The Raman data were acquired using RiverICon V 3.0.130327 software and were subsequently processed with the Skin Tools 2.0 (RiverD International B.V., Rotterdam, The Netherlands). The measurements taken on untreated skin served as a baseline and were subtracted from the drug concentration profiles. The estimation of SC thickness of each volunteer was based on the water/protein ratio across the scanned skin depth, calculated by the integration of the corresponding spectral peaks in the HWN region [47,48]. The SC thickness for TS was estimated as described by Kalia et al. (2001) [49], based on the TEWL values and the mass of SC removed from an untreated skin site. The TEWL values were recorded using an AquaFlux Model AF200 (Biox Systems Ltd., London, UK) instrument. The amount of SC removed was determined by measuring the protein content of the tape strips using a SquameScan^TM^ 850A (Heiland Electronic GmbH, Wetzlar, Germany) infrared densitometer [50,51]. The average SC thickness (h) for the volunteers was used to normalize the depth of each measurement (x) as a function of distance to the skin surface [23,26,44]. Data distributions were tested for normality using the Shapiro–Wilk test. Independent-samples t-test and one-way analysis of variance (ANOVA) with Tukey’s post hoc test were used to compare 2 and ≥3 normally distributed groups, respectively. A probability of *p* < 0.05 was considered to be statistically significant. The Wilcoxon Rank Sum and Kruskal–Wallis test were used to compare non-normally distributed datasets. All statistical analyses and figures were carried out using R (ver: 4.1.0) and RStudio (ver: 1.4.1717) [52].

## 3. Results and Discussion

### 3.1. In Vitro Permeation Studies

**Figure 1:**
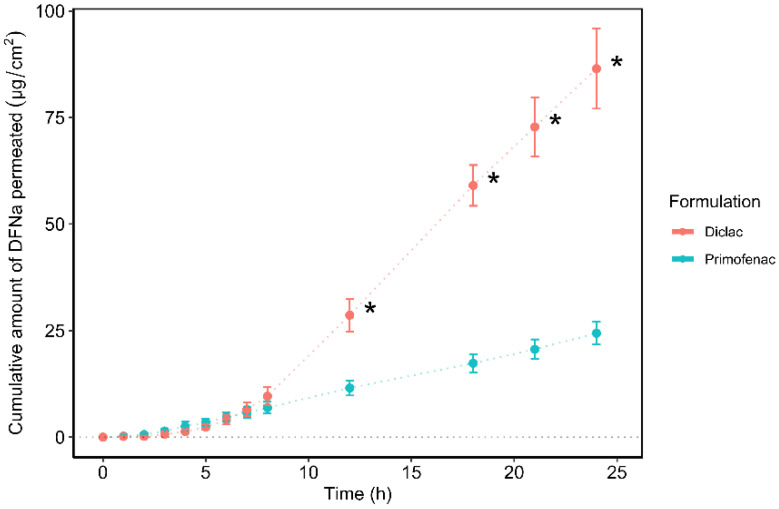
Cumulative permeation of DFNa over time for various commercially available formulations in human epidermis (*n* = 5; mean ± SD, * *p* < 0.05).

The permeation profiles of DFNa over 24 h, expressed as cumulative amounts (µg/cm^2^) are shown in Figure 1.

Diclac was found to outperform Primofenac in terms of promoting DFNa delivery to the skin over 24 h (86.5 ± 9.4 µg/cm^2^ and 24.4 ± 2.7 µg/cm^2^, respectively; *p* < 0.05). Additionally, the amounts of DFNa that permeated at earlier time intervals, i.e., 12 h, 18 h, and 21 h, were significantly higher for Diclac compared with Primofenac. The corresponding cumulative permeation values for these time points were: 28.6 ± 3.8 µg/cm^2^ vs. 11.5 ± 1.8 µg/cm^2^; *p* < 0.05; 59.1 ± 4.8 µg/cm^2^ vs. 17.3 ± 2.1 µg/cm^2^; *p* < 0.05; and 72.8 ± 6.9 µg/cm^2^ vs. 20.6 ± 2.3 µg/cm^2^; *p* < 0.05 for Diclac and Primofenac, respectively. The cumulative DFNa amounts that permeated the skin during the first 8 h were comparable for both formulations (*p* > 0.05). With regards to the rate of drug permeation, the Diclac gel promoted a significantly higher steady-state flux (J_ss_) value for the active agent over 24 h (4.8 ± 0.5 µg/cm^2/^h), compared to the Primofenac formulation (1.1 ± 0.1 µg/cm^2/^h; *p* < 0.05). It is worth noting that delivery from Primofenac was also associated with a shorter lag time (t_lag_) (1.7 ± 0.6 h), indicating that DFNa reached the J_ss_ more rapidly compared with Diclac (6.0 ± 0.8 h, *p* < 0.05). The steady-state flux of DFNa was determined from the slope of the linear portion of the cumulative amount of DFNa permeated per time unit (µg/cm^2/^h). The lag time was calculated from extrapolation of the linear portion to the *x*-axis intercept of the permeation profile [53].

The different performance of the two formulations may be attributed to their compositional differences, as shown in Table 1. More specifically, Diclac contains the long-chain lipophilic molecules decyl oleate (DO) and 2-octyldodecanol (OD). These solvents are an unsaturated fatty acid ester and aliphatic fatty alcohol, respectively, and they have been reported to be able to disturb the SC bilayers and promote drug permeation by lowering the diffusional resistance of the lipid domain [54]. Kakubari et al. (2006) examined the in vitro human skin permeation of formoterol fumarate from transdermal patches containing differing concentrations of OD, either 0.5 mg/cm^2^ or 1 mg/cm^2^ [55]. The incorporation of OD was reported to significantly enhance drug permeation compared to the control patch without OD. Additionally, a positive relationship was found between the extent of drug permeation and the concentration of OD in the transdermal patch, with a total 6.3-fold increase of cumulative drug permeation being reported when the OD concentration was raised from 0 to 1.0 mg/cm^2^. More recently, Ameen and Michniak-Kohn (2019) investigated the impact of several penetration enhancers, including OD and decyl oleate (DO), on the delivery of galantamine across dermatomed human skin ex vivo [56]. A penetration enhancer at 5% (*w*/*w*) of dry polymer weight was loaded into galantamine patches, and drug permeability was assessed against a control patch that contained no penetration enhancer. The incorporation of either DO or OD into the patch was reported to significantly increase the steady-state transdermal flux values by a factor of 2.0 and 1.7, respectively, compared to the control. An increase of the cumulative amount of the drug permeated and a 30 min reduction in the lag time were also reported for both DO and OD, indicating a promotion of drug diffusivity through the skin. In a different study, Montenegro et al. (2011) prepared a series of microemulsions that contained 5% of either OD, DO, or medium chain triglycerides (MCT) and examined the impact of these solvents on skin delivery of a model active agent, octyl-methoxycinnamate [57]. These researchers conducted 24 h Franz-type permeation studies in excised human epidermis under infinite dose conditions (500 µL). Overall, the presence of OD or DO in the formulations was found to promote greater cumulative permeation of the active agent (44.1 ± 8.8 µg/cm^2^ and 12.9 ± 2.3 µg/cm^2^, respectively) compared with the MCT-containing emulsions (2.0 ± 0.5 µg/cm^2^). Additionally, inclusion of either OD or DO resulted in greater flux values (2.4 ± 0.5 µg/cm^2^/h and 0.7 ± 0.1 µg/cm^2^/h, respectively) than the MCT (0.1 ± 0.03 µg/cm^2^/h). Here, the greater efficacy of Diclac in terms of promoting DFNa topical permeation is likely attributed to the presence of DO and OD in this formulation compared with Primofenac (Table 1).

Additionally, several studies in the scientific literature have reported that combinations of solvents with differing physicochemical properties resulted in synergistic enhancement of drug permeation [58,59,60,61,62,63]. Brinkmann and Muller-Goymann (2003) investigated the effect of isopropanol (IPA) and a long-chain fatty acid ester, namely isopropyl myristate (IPM), on the permeation of hydrocortisone (HC) across isolated human SC [64]. Various ointment formulations containing IPA and/or IPM were examined, while a control formulation without these solvents was also used. These workers also conducted differential scanning calorimetry and wide angle and small angle X-ray diffraction studies to assess the effects of these solvents on the human SC following treatment with IPM, IPA, or a combination of both for 30 min. Overall, the formulations containing both solvents were found to deliver significantly higher amounts of HC compared with the other formulations tested. The authors suggested that the combination of IPA with IPM resulted in a stronger fluidization and disruption of intercellular lipids than IPA alone, thus promoting a synergistic penetration enhancement. More recently, Parisi et al. (2016) reported that combinations of IPA with other ingredients (propylene glycol, glycerol, or PEG-200) resulted in a significant increase of permeation of hexamidine diisethionate across porcine skin in vitro compared with the individual solvents under infinite dose conditions [65]. In the present study, both formulations contained the short-chain alcohol IPA together with additional components (Table 1). The different performance of the two formulations in terms of promoting DFNa delivery might be attributed to synergistic effects among the various components of Diclac compared to Primofenac.

### 3.2. In Vivo Studies

#### 3.2.1. Confocal Raman Spectroscopy

**Figure 2:**
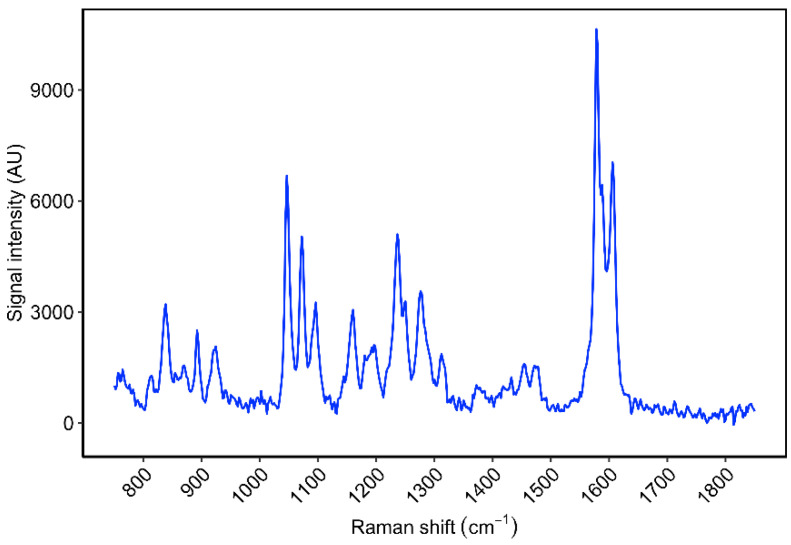
Reference Raman spectrum of DFNa in PG solution (200 mg/mL), using a 10 s exposure time, mean of 10 frames.

The reference spectrum of DFNa is shown in Figure 2. The Raman spectrum of DFNa is consistent with previous reports in the literature [37,66].

The measured SC thickness values for volunteers 1 and 2 were 16.3 ± 2.3 µm and 17.4 ± 3.5 µm, respectively. The use of CRS for measuring human SC thickness in vivo has been validated in prior studies in the literature, with CRS results being reported to be in agreement with findings from different methodologies, such as optical coherence tomography and confocal reflectance microscopy [48,67].

**Figure 3:**
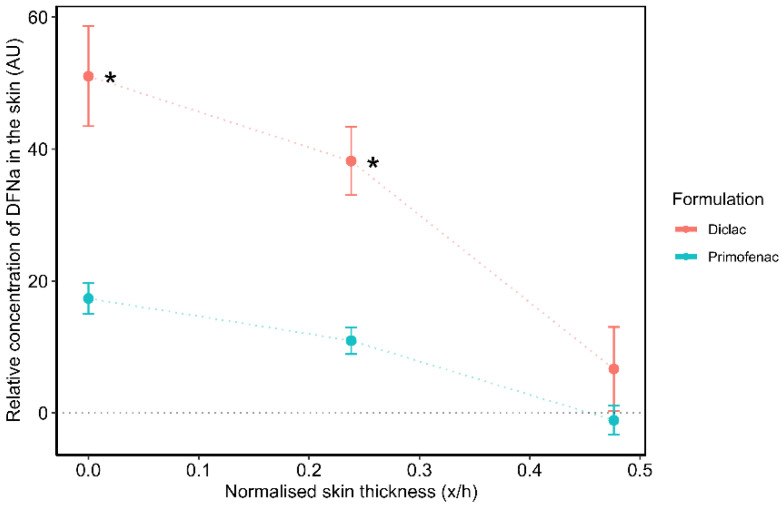
Depth profiles of DFNa (AU) across the SC estimated by CRS following a 30 min application of formulations in vivo (mean ± SEM of 2 subjects; *n* ≥ 6 replicates per subject; * *p* < 0.05).

The DFNa concentration profiles across the volar forearm skin of the volunteers for the two formulations are shown below in Figure 3.

Both formulations were found to promote DFNa penetration to the SC over 30 min. Diclac was found to deliver significantly higher amounts of the drug to the upper 4 µm of the skin compared with Primofenac (*p* < 0.05). This depth corresponded to a depth interval ∼ 0.25 x/h of the average SC thickness. The DFNa signal intensity values for Diclac and Primofenac at this depth were 38.2 ± 5.2 AU and 10.9 ± 2.0 AU, respectively. Diclac was additionally found to promote DFNa permeation to 8 µm, i.e., ∼ 0.5 x/h depth interval, while no DFNa signal was detected at this depth for Primofenac. Neither formulation delivered DFNa to SC layers deeper than 0. 5 x/h. With regards to the total amount of DFNa penetrated in vivo, the area under the CRS depth profile curves (AUC) was used as a measure of the DFNa skin uptake for every formulation [26,28,44,46]. The AUC was calculated based on the composite trapezoidal rule [68]. The AUC values, expressed as signal intensity units (AU) multiplied by the normalized depth (x/h), were 16.0 ± 2.9 and 4.5 ± 1.0 for Diclac and Primofenac, respectively. These values indicated that Diclac resulted in a ~3.5 times greater total SC uptake of DFNa compared with Primofenac. This is consistent with the estimated extent of DFNa penetration to the upper SC based on the observed signal intensity values at the 4 µm SC depth.

#### 3.2.2. Tape Stripping

**Figure 4:**
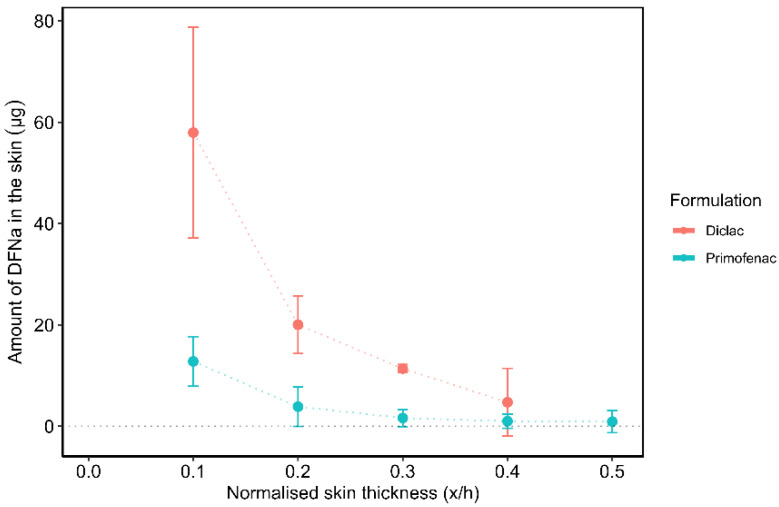
Amount of DFNa (µg) removed by TS across the SC following a 30 min application of formulations (mean ± SD, *n* = 2 subjects).

The concentration profiles determined by TS are shown in Figure 4. The SC thickness for the skin sites of the volunteers estimated by TS and TEWL was 5.2 ± 1.0 µm and 6.1 ± 0.2 µm. Overall, Diclac was found to outperform Primofenac in terms of promoting DFNa delivery to the skin. This difference in the efficacy of the two formulations is consistent with the results obtained by CRS (Figure 3).

DFNa content at 0.1 x/h depth interval was 4.5 times greater for Diclac compared to Primofenac (58.0 ± 20.8 µg vs. 12.8 ± 4.9 µg, respectively). The amounts of DFNa extracted from the 0.2 x/h depth interval were 20.0 ± 5.7 µg and 3.9 ± 0.4 µg for Diclac and Primofenac, respectively. The total DFNa amounts estimated from all 20 tape strips were 94.1 ± 22.6 µg for Diclac and 20.2 ± 7.0 µg for Primofenac. DFNa was found to penetrate to a maximum depth interval of ~0.5 x/h of the SC thickness. The depth of DFNa penetration is in agreement with the data reported for the same formulations when evaluated by CRS (Figure 3). This observation is consistent with a recent report in the literature, where skin absorption of an active agent, retinol, was examined by TS and CRS [69]. These researchers conducted individual experiments of TS and CRS to measure the ex vivo skin disposition of retinol in porcine skin, an acceptable surrogate for human skin [70]. Although slight differences in the shape of the penetration profiles were reported for the two methods, the maximum penetration depth of the active agent was found to be 15 µm for both CRS and TS, with negligible retinol concentrations detected deeper than 16 µm of skin depth.

### 3.3. In Vitro–In Vivo Correlations

Table 2 shows the relevant permeation parameters for all methods examined. The cumulative drug permeation for Diclac at 24 h in vitro (Q_24_) is an index of formulation efficacy for delivery of the active agent. The total skin absorption values of compounds have been previously used as a metric for comparison of various methodologies in numerous reports in the literature [4,26,28,46,71]. In the present work, the cumulative DFNa permeation for Diclac was found to be 3.6 times greater compared with the corresponding Q_24_ value for Primofenac. These findings are very similar to the results obtained by CRS for either the total skin uptake of the drug (AUC_Diclac_/AUC_Primofenac_ = 3.6) or the signal intensity values measured at 0.25 x/h depth interval (CRS Intensity_Diclac_/CRS Intensity_Primofenac_ = 3.5), indicating good consistency between the two methods (Table 2). These findings demonstrate the sensitivity of CRS to the differences between the two formulations in a similar manner as for the IVPT model.

The findings of the present study are in agreement with previous reports in the literature, where the CRS signal detection of active agents in the upper SC layers in vivo has been correlated with data from in vitro permeation studies. Mohammed et al. (2014) showed that Raman signal intensity of niacinamide (NIA) at a depth of 4 µm in the SC in vivo was linearly proportional to the corresponding flux values of the active agent measured by in vitro permeation studies [27]. Values for the correlation coefficients (R^2^) were found to be 0.96 and 0.91 for NIA flux values greater or lower than 10 µg/cm^2^/h, respectively. More recently, an excellent correlation (R^2^ = 0.98) was also reported between the cumulative permeation of NIA in vitro (Q_24_) and the amount of active agent taken up by the skin for a depth of 2 µm in vivo [28]. In the same study, the total amount of NIA taken up by the skin was additionally calculated by numerical integration of the depth profiles curves (AUC), assuming a SC thickness of 15 µm. A Pearson correlation value of R^2^ = 0.94 was reported between the total uptake in vivo and the cumulative amounts of NIA permeated in vitro for the various formulations. The same approach was also used by Patel et al. (2021), who examined the drug, ibuprofen, and vehicle disposition with the in vitro permeation model and CRS in vivo studies [44]. An excellent correlation was reported (R^2^ = 0.90) following the linear regression of the cumulative permeation of ibuprofen in vitro (Q_24_) and the corresponding amount taken up by the SC measured by CRS in vivo. The area under the Raman signal curve was also used as a measure of the total NIA uptake by Zhang et al. (2021). The CRS AUC data were found to correlate very well with the cumulative amounts of NIA permeated in vitro, with a correlation coefficient (R^2^) of 0.84. With regards to the TS methodology, the total amount of DFNa that penetrated the SC for Diclac was found to be 4.7 times higher than for Primofenac. This 4.7-fold difference in performance between the two formulations is greater compared with the cumulative permeation results obtained by the IVPT method (3.6-fold difference in Q_24_ values; Table 2). This may be attributed to several previously reported experimental variabilities that are associated with TS. These include the pressure applied to the strip prior to stripping, the duration of contact time with the SC, and the velocity of tape removal during the procedure. In addition, Van der Molen et al. (1997) investigated the effect that skin furrows can have on the results of TS. These researchers applied 2 mg/cm^2^ of a TiO_2_ containing formulation to human skin in vitro, which was subsequently tape-stripped 15 times [72]. Subsequently, X-ray microanalysis and scanning electron microscopy were used to examine the titanium residues in the stripped skin. A persistent presence of titanium in the rims of skin furrows was reported, and microscopic visualization of the stripped skin areas showed that furrows were still evident even after removing 20 tape strips. The researchers suggested that some parts of the SC may not be completely removed by the TS procedure. The work suggested that residual formulation could accumulate in furrows that are not collected by the tape strips and thus would contribute to significant variability in skin uptake results of active agents.

## 4. Conclusions

The present work examined the percutaneous delivery of DFNa from two marketed formulations with different compositions, both in vitro and in vivo. This is the first study to examine the capability of CRS to profile drug disposition from complex commercially available dermatological drug products. Overall, results from all methodologies examined showed that Diclac outperformed Primofenac in terms of promoting DFNa delivery to the skin (*p* < 0.05). The in vitro cumulative amounts of the drug permeated at 24 h from Diclac (86.5 ± 9.4 µg/cm^2^) were 3.6 times greater than for Primofenac (24.4 ± 2.7 µg/cm^2^). The performance of these formulations estimated by CRS in vivo was in agreement with the IVPT results. Both total skin uptake of DFNa (AUC) and the signal intensity of the drug detected in the upper SC (0.25 x/h of the average SC thickness) were found to be 3.5–3.6 times greater for Diclac than for Primofenac. With regards to TS data, a 4.7-fold greater efficacy of Diclac was found relative to Primofenac in terms of promoting DFNa skin delivery. The findings of this study demonstrate that CRS is a methodology that can be used to discriminate between and evaluate complex topical formulations. Additionally, the non-invasive nature of CRS coupled with its capability of monitoring drug permeation in real time underline the utility of this approach for evaluation of topical bioequivalence. Future work will further examine the sensitivity and reproducibility of CRS by using additional drugs and formulations. Studies are ongoing to include a greater number of time points and subjects that will enable a comprehensive dermatopharmacokinetic analysis.

## Figures and Tables

**Table 1:** Ingredients of the topical formulations, Diclac^®^ and Primofenac^®^.

Diclac^®^ Lipogel 10 mg/g	Primofenac^®^ Emulsion gel 1%
DFNa	DFNa
RRR-α-tocopherol	cetyl alcohol
carbomer 980 NF	methyl-4-hydroxuybenzoate
decyl oleate	propyl-4-hydroxybenzoate
2-octyldodecanol	isopropyl alcohol
Lecithin	glycerol
ammonium hydroxide 10%	polyacrylic acid (Carbomer)
disodium edetate	medium-chain triglycerides
perfume oil ’Vert de Creme’	macrogol cetostearyl ether
isopropyl alcohol	purified water
purified water	

**Table 2:** Comparison of skin permeation parameters for Diclac^®^ and Primofenac^®^, measured by the in vitro permeation model, confocal Raman spectroscopy in vivo and tape stripping in vivo.

Formulation	In vitro			In vivo		
	Q_24_ (µg/cm^2^)	J_ss_ (µg/cm^2^/h)	t_lag_ (h)	AUC_CRS_ (AU)	CRS intensity at 0.25 x/h (AU)	Q_TS_ (µg)
Diclac	86.5 ± 9.4	4.8 ± 0.5	6.0 ± 0.8	16.0 ± 2.9	38.2 ± 5.2	94.1 ± 22.6
Primofenac	24.4 ± 2.7	1.1 ± 0.1	1.7 ± 0.6	4.5 ± 1.0	10.9 ± 2.0	20.2 ± 7.0

Q_24_: cumulative in vitro permeation at 24 h; J_ss_: steady-state flux; t_lag_: lag time; AUC_CRS_: area under the CRS depth profiles curves; Q_TS_: total amount of DFNa extracted by tape stripping.

## Data Availability

Not applicable.

## References

[1] Ghosh P., Raney S., Luke M. (2021). Evaluation of Cutaneous Pharmacokinetics: The Past, the Present and the Future.

[2] Raney S.G., Franz T.J., Lehman P.A., Lionberger R., Chen M.-L. (2015). Pharmacokinetics-Based Approaches for Bioequivalence Evaluation of Topical Dermatological Drug Products. Clin. Pharmacokinet..

[3] Kezic S. (2008). Methods for measuring in-vivo percutaneous absorption in humans. Hum. Exp. Toxicol..

[4] Lehman P.A., Raney S.G., Franz T.J. (2011). Percutaneous Absorption in Man: In vitro-in vivo Correlation. Skin Pharmacol. Physiol..

[5] Dupuis D., Rougier A., Roguet R., Lotte C., Kalopissis G. (1984). In vivo Relationship between Horny Layer Reservoir Effect and Percutaneous Absorption in Human and Rat. J. Investig. Dermatol..

[6] Zsikó S., Csányi E., Kovács A., Budai-Szűcs M., Gácsi A., Berkó S. (2019). Methods to Evaluate Skin Penetration In Vitro. Sci. Pharm..

[7] Keurentjes A.J., Jakasa I., Kezic S. (2021). Research Techniques Made Simple: Stratum Corneum Tape Stripping. J. Investig. Dermatol..

[8] Bashir S.J., Chew A.L., Anigbogu A., Dreher F., Maibach H.I. (2001). Physical and physiological effects of stratum corneum tape stripping. Ski. Res. Technol..

[9] Jacobi U., Meykadeh N., Sterry W., Lademann J. (2003). Effect of the vehicle on the amount of stratum corneum removed by tape stripping. JDDG J. Dtsch. Dermatol. Ges..

[10] Löffler H., Dreher F., Maibach H.I. (2004). Stratum corneum adhesive tape stripping: Influence of anatomical site, application pressure, duration and removal. Br. J. Dermatol..

[11] Lademann J., Jacobi U., Surber C., Weigmann H.J., Fluhr J.W. (2009). The tape stripping procedure—Evaluation of some critical parameters. Eur. J. Pharm. Biopharm..

[12] US Food and Drug Administration (2002). Draft Guidance for Industry on Topical Dermatological Drug Product NDAs and ANDAs-In Vivo Bioavailability, Bioequivalence, In Vitro Release and Associated Studies; Withdrawal. Fed. Regist..

[13] Goh C.F., Moffat J.G., Craig D.Q.M., Hadgraft J., Lane M.E. (2019). Monitoring Drug Crystallization in Percutaneous Penetration Using Localized Nanothermal Analysis and Photothermal Microspectroscopy. Mol. Pharm..

[14] Hadgraft J., Lane M.E. (2016). Drug crystallization—Implications for topical and transdermal delivery. Expert Opin. Drug Deliv..

[15] Caspers P.J., Lucassen G.W., Wolthuis R., Bruining H.A., Puppels G.J. (1998). In vitro and in vivo Raman spectroscopy of human skin. Biospectroscopy.

[16] Caspers P.J., Bruining H.A., Puppels G.J., Lucassen G.W., Carter E.A. (2001). In Vivo Confocal Raman Microspectroscopy of the Skin: Noninvasive Determination of Molecular Concentration Profiles. J. Investig. Dermatol..

[17] Lieber C., Mahadevan-Jansen A. (2007). Development of a handheld Raman microspectrometer for clinical dermatologic applications. Opt. Express.

[18] Franzen L., Windbergs M. (2015). Applications of Raman spectroscopy in skin research—From skin physiology and diagnosis up to risk assessment and dermal drug delivery. Adv. Drug Deliv. Rev..

[19] van der Pol A., Caspers P.J. (2019). Confocal Raman Spectroscopy for In Vivo Skin Hydration Measurement. Handbook of Cosmetic Science and Technology.

[20] Pena A.-M., Chen X., Pence I.J., Bornschlögl T., Jeong S., Grégoire S., Luengo G.S., Hallegot P., Obeidy P., Feizpour A. (2020). Imaging and quantifying drug delivery in skin—Part 2: Fluorescence andvibrational spectroscopic imaging methods. Adv. Drug Deliv. Rev..

[21] Pudney P.D.A., Mélot M., Caspers P.J., van der Pol A., Puppels G.J. (2007). An In Vivo Confocal Raman Study of the Delivery of Trans-Retinol to the Skin. Appl. Spectrosc..

[22] Mélot M., Pudney P.D.A., Williamson A.-M., Caspers P.J., Van Der Pol A., Puppels G.J. (2009). Studying the effectiveness of penetration enhancers to deliver retinol through the stratum cornum by in vivo confocal Raman spectroscopy. J. Control Release.

[23] Mateus R., Abdalghafor H., Oliveira G., Hadgraft J., Lane M.E. (2013). A new paradigm in dermatopharmacokinetics—Confocal Raman spectroscopy. Int. J. Pharm..

[24] Herkenne C., Naik A., Kalia Y.N., Hadgraft J., Guy R.H. (2006). Pig Ear Skin ex Vivo as a Model for in Vivo Dermatopharmacokinetic Studies in Man. Pharm. Res..

[25] Santos P., Watkinson A.C., Hadgraft J., Lane M.E. (2011). Enhanced permeation of fentanyl from supersaturated solutions in a model membrane. Int. J. Pharm..

[26] Mateus R., Moore D.J., Hadgraft J., Lane M.E. (2014). Percutaneous absorption of salicylic acid—In vitro and in vivo studies. Int. J. Pharm..

[27] Mohammed D., Matts P.J., Hadgraft J., Lane M.E. (2014). In Vitro–In Vivo Correlation in Skin Permeation. Pharm. Res..

[28] Iliopoulos F., Caspers P.J., Puppels G.J., Lane M.E. (2020). Franz Cell Diffusion Testing and Quantitative Confocal Raman Spectroscopy: In Vitro-In Vivo Correlation. Pharmaceutics.

[29] Skelly J.P., Shah V.P., Maibach H.I., Guy R.H., Wester R.C., Flynn G., Yacobi A. (1987). FDA and AAPS Report of the Workshop on Principles and Practices of In Vitro Percutaneous Penetration Studies: Relevance to Bioavailability and Bioequivalence. Pharm. Res..

[30] Franz T.J., Lehman P.A., Raney S.G. (2009). Use of Excised Human Skin to Assess the Bioequivalence of Topical Products. Skin Pharmacol. Physiol..

[31] Shin S.H., Thomas S., Raney S.G., Ghosh P., Hammell D.C., El-Kamary S.S., Chen W.H., Billington M.M., Hassan H.E., Stinchcomb A.L. (2018). In vitro–in vivo correlations for nicotine transdermal delivery systems evaluated by both in vitro skin permeation (IVPT) and in vivo serum pharmacokinetics under the influence of transient heat application. J. Control Release.

[32] Shin S.H., Rantou E., Raney S.G., Ghosh P., Hassan H., Stinchcomb A. (2020). Cutaneous Pharmacokinetics of Acyclovir Cream 5% Products: Evaluating Bioequivalence with an In Vitro Permeation Test and an Adaptation of Scaled Average Bioequivalence. Pharm. Res..

[33] Pyatski Y., Zhang Q., Mendelsohn R., Flach C.R. (2016). Effects of permeation enhancers on flufenamic acid delivery in Ex vivo human skin by confocal Raman microscopy. Int. J. Pharm..

[34] Goh C.F., Lane M.E. (2014). Formulation of diclofenac for dermal delivery. Int. J. Pharm..

[35] Thomas G.J., Herranz P., Cruz S.B., Parodi A. (2019). Treatment of actinic keratosis through inhibition of cyclooxygenase-2: Potential mechanism of action of diclofenac sodium 3% in hyaluronic acid 2.5. Dermatol. Ther..

[36] Goh C.F., Boyd B.J., Craig D.Q.M., Lane M.E. (2020). Profiling of drug crystallization in the skin. Expert Opin. Drug Deliv..

[37] Goh C.F., Craig D.Q.M., Hadgraft J., Lane M.E. (2017). The application of ATR-FTIR spectroscopy and multivariate data analysis to study drug crystallisation in the stratum corneum. Eur. J. Pharm. Biopharm..

[38] ICH Harmonised Tripartite Validation of analytical procedures: Text and methodology Q2 (R1). Proceedings of the International Conference on Harmonization.

[39] Organization for Economic Cooperation Development (2004). Test No. 428: Skin absorption: In vitro method. OECD Guidelines for the Testing of Chemicals, Section 4.

[40] Organization for Economic Cooperation Development (2004). OECD Guidance document for the conduct of skin absorption studies. OECD Series on Testing and Assessment.

[41] Iliopoulos F., Chapman A., Lane M.E. (2021). A comparison of the in vitro permeation of 3-O-ethyl-l-ascorbic acid in human skin and in a living skin equivalent (LabSkin™). Int. J. Cosmet. Sci..

[42] Oliveira G., Hadgraft J., Lane M.E. (2012). The influence of volatile solvents on transport across model membranes and human skin. Int. J. Pharm..

[43] The RefData Foundation (2020). Primofenac^®^ Emulsions-Gel Patient Information. Medicinal Product Information Search Platform (AIPS) for Authorised Human Medicines. https://www.swissmedicinfo.ch/.

[44] Patel A., Iliopoulos F., Caspers P.J., Puppels G.J., Lane M.E. (2021). In Vitro–In Vivo Correlation in Dermal Delivery: The Role of Excipients. Pharmaceutics.

[45] Mohammed D., Matts P.J., Hadgraft J., Lane M.E. (2011). Depth profiling of stratum corneum biophysical and molecular properties. Br. J. Dermatol..

[46] Zhang Y., Kung C.-P., Iliopoulos F., Sil B.C., Hadgraft J., Lane M.E. (2021). Dermal Delivery of Niacinamide—In Vivo Studies. Pharmaceutics.

[47] Caspers P., Lucassen G., Bruining H., Puppels G. (2000). Automated depth-scanning confocal Raman microspectrometer for rapid in vivo determination of water concentration profiles in human skin. J. Raman Spectrosc..

[48] Crowther J.M., Sieg A., Blenkiron P., Marcott C., Matts P.J., Kaczvinsky J.R., Rawlings A.V. (2008). Measuring the effects of topical moisturizers on changes in stratum corneum thickness, water gradients and hydration in vivo. Br. J. Dermatol. (1951).

[49] Kalia Y.N., Alberti I., Naik A., Guy R.H. (2001). Assessment of Topical Bioavailability in vivo: The Importance of Stratum corneum Thickness. Skin Pharmacol. Physiol..

[50] Voegeli R., Heiland J., Doppler S., Rawlings A.V., Schreier T. (2007). Efficient and simple quantification of stratum corneum proteins on tape strippings by infrared densitometry. Skin Res. Technol..

[51] Mohammed D., Yang Q., Guy R.H., Matts P.J., Hadgraft J., Lane M.E. (2012). Comparison of gravimetric and spectroscopic approaches to quantify stratum corneum removed by tape-stripping. Eur. J. Pharm. Biopharm..

[52] Team R. (2021). Integrated Development Environment for R.

[53] Finnin B., Walters K.A., Franz T.J. (2012). In vitro skin permeation methodology. Transdermal and Topical Drug Delivery: Principles and Practice.

[54] Loth H. (1991). Vehicular influence on transdermal drug penetration. Int. J. Pharm..

[55] Kakubari I., Sasaki H., Takayasu T., Yamauchi H., Takayama S., Takayama K. (2006). Effects of ethylcellulose and 2-octyldodecanol additives on skin permeation and irritation with ethylene-vinyl acetate copolymer matrix patches containing formoterol fumarate. Biol. Pharm. Bull..

[56] Ameen D., Michniak-Kohn B. (2019). Development and in vitro evaluation of pressure sensitive adhesive patch for the transdermal delivery of galantamine: Effect of penetration enhancers and crystallization inhibition. Eur. J. Pharm. Biopharm..

[57] Montenegro L., Carbone C., Puglisi G. (2011). Vehicle effects on in vitro release and skin permeation of octylmethoxycinnamate from microemulsions. Int. J. Pharm..

[58] Mitriaikina S., Muller-Goymann C.C. (2007). Synergetic effects of isopropyl alcohol (IPA) and isopropyl myristate (IPM) on the permeation of betamethasone-17-valerate from semisolid Pharmacopoeia bases. J. Drug Deliv. Sci. Technol..

[59] Lane M.E., Santos P., Watkinson A.C., Hadgraft J. (2012). Passive Skin Permeation Enhancement. Topical and Transdermal Drug Delivery.

[60] Lane M.E. (2013). Skin penetration enhancers. Int. J. Pharm..

[61] Iliopoulos F., Sil B.C., Moore D.J., Lucas R.A., Lane M.E. (2019). 3-O-ethyl-l-ascorbic acid: Characterisation and investigation of single solvent systems for delivery to the skin. Int. J. Pharm. X.

[62] Iliopoulos F., Hossain A.S.M.M.A., Sil B.C., Moore D.J., Lucas R.A., Lane M.E. (2020). Topical Delivery of 3-O-ethyl l-ascorbic Acid from Complex Solvent Systems. Sci. Pharm..

[63] Kung C.-P., Zhang Y., Sil B.C., Hadgraft J., Lane M.E., Patel B., McCulloch R. (2020). Investigation of binary and ternary solvent systems for dermal delivery of methadone. Int. J. Pharm..

[64] Brinkmann I., Müller-Goymann C.C. (2003). Role of Isopropyl Myristate, Isopropyl Alcohol and a Combination of Both in Hydrocortisone Permeation across the Human Stratum corneum. Skin Pharmacol. Physiol..

[65] Parisi N., Paz-Alvarez M., Matts P.J., Lever R., Hadgraft J., Lane M.E. (2016). Topical delivery of hexamidine. Int. J. Pharm..

[66] Zhang Q., Flach C.R., Mendelsohn R., Page L., Whitson S., Boncheva Bettex M. (2020). Visualization of Epidermal Reservoir Formation from Topical Diclofenac Gels by Raman Spectroscopy. J. Pain Res..

[67] Böhling A., Bielfeldt S., Himmelmann A., Keskin M., Wilhelm K.P. (2014). Comparison of the stratum corneum thickness measured in vivo with confocal Raman spectroscopy and confocal reflectance microscopy. Skin Res. Technol..

[68] Signorell A., Aho K., Alfons A., Anderegg N., Aragon T., Arachchige C., Arppe A., Baddeley A., Barton K., Bolker B. DescTools: Tools for Descriptive Statistics. https://cran.r-project.org/package=DescTools.

[69] Krombholz R., Fressle S., Lunter D. (2022). Ex vivo—In vivo correlation of retinol stratum corneum penetration studies by confocal Raman microspectroscopy and tape stripping. Int. J. Cosmet. Sci..

[70] Barbero A.M., Frasch H.F. (2009). Pig and guinea pig skin as surrogates for human in vitro penetration studies: A quantitative review. Toxicol. Vitr..

[71] van de Sandt J.J.M., Meuling W.J.A., Elliott G.R., Cnubben N.H.P., Hakkert B.C. (2000). Comparative in Vitro–in Vivo Percutaneous Absorption of the Pesticide Propoxur. Toxicol. Sci..

[72] van der Molen R.G., Spies F., van ‘t Noordende J.M., Boelsma E., Mommaas A.M., Koerten H.K. (1997). Tape stripping of human stratum corneum yields cell layers that originate from various depths because of furrows in the skin. Arch. Dermatol. Res..

